# Genomic Expansion and Adaptation in a Parasitoid Wasp *Eretmocerus hayati* (Hymenoptera: Aphelinidae)

**DOI:** 10.3390/insects17040369

**Published:** 2026-03-31

**Authors:** Yuwei Zhong, Yunyun Fan, Ruoxin Ruan, Dujun Xi, Huifeng Luo, Ce Li, Hui Liu, Yinquan Liu

**Affiliations:** 1 Institute of Horticulture, Hangzhou Academy of Agricultural Sciences, Hangzhou 310024, China; ywzhong@zju.edu.cn (Y.Z.);; 2 Ministry of Agriculture and Rural Affairs Key Laboratory of Molecular Biology of Crop Pathogens and Insects, Zhejiang Key Laboratory of Biology and Ecological Regulation of Crop Pathogens and Insects, Institute of Insect Sciences, Zhejiang University, Hangzhou 310058, China; 3 Zhejiang Tianmushan National Nature Reserve Administration, Hangzhou 311311, China; 4 Shenyang Key Laboratory of Surveillance and Management for Vegetable Diseases and Insect Pests, College of Plant Protection, Shenyang Agricultural University, Shenyang 110866, China

**Keywords:** parasitoid wasp, genome evolution, *Eretmocerus hayati*, Aphelinidae, LTR insertion, biological control

## Abstract

The parasitic wasps of Aphelinidae (Hymenoptera) are a group of insects with significant biological control value. However, their genomic evolution and ecological adaptation mechanisms remain unclear. In this study, we focused on the genome analysis of *Eretmocerus hayati* and performed a comparative analysis with four other species from Aphelinidae. Our results indicated that the divergence time of Aphelinidae was approximately 119.9 million years ago. In *Er. hayati*, gene families related to energy metabolism and humoral immunity have significantly expanded, which may be associated with the high metabolic demands of its small body size and the immune adaptation strategies resulting from its unique parasitic methods. Additionally, genes involved in DNA replication and recombination have undergone positive selection in the ancestral branch of Aphelinidae species. Compared with 23 species within nine families in the Chalcidoidea superfamily studied here, the genome size of *Er. hayati* is the largest. The analysis of repetitive sequences revealed a recent burst of long terminal repeat (LTR) sequence insertions in the genome of *Er. hayati*. Our study indicates the evolutionary characteristics of *Er. hayati* in terms of gene family evolution, chromosomal collinearity, and transposon dynamics, providing a theoretical basis for understanding the environmental adaptation and biological control applications.

## Simple Summary

Parasitic wasps have attracted growing attention in biological control research. Whole-genome analysis enables researchers to discover details that were previously undetectable using experimental methods, thus enhancing our understanding of the biology and evolution of specific species. In this study, we conducted a genomic analysis of *Eretmocerus hayati*, an agriculturally important parasitoid wasp, revealing key features related to gene family evolution, chromosomal synteny, and transposable element dynamics. These findings provide a theoretical basis for understanding the evolutionary adaptations of this beneficial insect.

## 1. Introduction

Parasitoid wasps represent one of the most species-rich groups of insects and play important roles in biological control and co-evolution studies [1]. With the widespread adoption of high-throughput sequencing technologies, many parasitoid wasp genomes have been assembled with high quality, providing a data foundation for in-depth analysis of genomic characteristics [2]. Genome analysis of parasitoid wasps is critical for understanding parasitic adaptability and genomic evolution. Comparative genomics has revealed the evolutionary diversity of venom components across parasitoid wasps. Studies have demonstrated significant variations in the host adaptation of wasp venom, with some venom genes undergoing positive selection [2,3]. Using genomic, transcriptomic, and proteomic data, researchers can comprehensively identify genes specifically expressed in venom glands, successfully characterizing dozens of venom proteins with potential functions and elucidating the molecular interaction mechanisms between parasitic wasps and their hosts [4,5]. Beyond venom systems, genomic analyses have also uncovered other evolutionary adaptations. Notably, several studies have revealed the integration of viruses into parasitoid genomes, which suppress host immune systems and ensure reproductive success [6,7,8,9]. Additionally, gene family expansion has been associated with reproductive adaptations. In *Diachasma alloeum*, genomic evidence indicates that gene duplications are associated with asexual reproduction strategies, highlighting the mechanistic pathways that enable female offspring production without fertilization [10,11].

Genomes of parasitoid wasps exhibit substantial size variation, ranging from 180 Mb in *Megaphragma amalphitanum* [12] to 878 Mb in *Megastigmus duclouxiana* [13]. The expansion and elimination of transposable elements (TEs) are primary drivers of this genome size divergence [14]. For example, the genomes of two parasitic wasps in the genus *Anastatus* (*A. japonicus* and *A. fulloi*), which are notably larger than those of other parasitoid species, show evidence of recent bursts of long terminal repeat (LTR) retrotransposon activity, suggesting that specific evolutionary events may facilitate genome size changes [14]. Similarly, studies on other important groups such as the Braconidae have also confirmed the presence of highly divergent TE families, which constitute a major contributor to genomic complexity [15].

*Eretmocerus hayati* (Hymenoptera: Aphelinidae) is an important parasitoid of *Bemisia tabaci* (Hemiptera: Aleyrodidae), a globally significant pest whitefly [16,17,18]. Field releases of *Er. hayati* have proven effective in suppressing whitefly outbreaks [19,20,21]. To date, five species of the family Aphelinidae, including *Er. Hayati,* have been assembled at the chromosomal level. In this study, we conducted a comparative genomic analysis of *Er. hayati* and the other four species using their available genome assemblies and gene annotations [22]. A phylogenetic tree was constructed to infer their evolutionary history. Our analysis revealed several significantly expanded gene families in *Er. hayati* that are involved in chromosome organization and immune responses. Positive selection analysis revealed that chromosome recombination-related genes in *Er. hayati* have undergone rapid evolution. Given the observed large genome size, we conducted an ancestral state reconstruction analysis, revealing a trend of genome expansion. Further analysis indicated that the extensive LTR sequence insertions might drive genomic enlargement in *Er. hayati*.

## 2. Materials and Methods

### 2.1. Comparative Genomics and Phylogenetic Analysis

The chromosomal-level genome assembly of *Er. hayati* was generated in our previous studies using Hi-C sequencing technology [22]. Genome annotation was subsequently performed utilizing 28 transcriptome datasets (Supplementary Table S1) from different larval stages and environmental conditions [22]. To perform comparative genomic analysis, we obtained genome annotation data of the representative species in Hymenoptera from the National Center for Biotechnology Information (NCBI) and InsectBase database (https://insect-genome.com) (Supplementary Table S2).

The annotated protein sequences of *Er. hayati* and 39 other hymenopteran species were used for comparative genomics and phylogenomic analyses. The ingroup comprised 37 Apocrita species: 30 parasitic wasps, including 24 Chalcidoidea species, two Cynipoidea species and four Ichneumonoidea species; and seven non-parasitic wasps, comprising two Apoidea species, two Formicoidea species, two Vespoidea species and one Chrysidoidea species. The outgroups comprised three hymenopteran species, one each from Orussoidea, Cephoidea, and Tenthredinoidea.

The longest transcripts of each gene were retained for further analysis. OrthoFinder (v2.5.4) [23,24] with the parameter “-m MSA” was used to identify orthologous and paralogous gene groups. To reconstruct the phylogeny of these 40 hymenopteran species, MAFFT (v7.471) [25] was used with the “-auto” parameter to independently align multiple genes in each single-copy gene group generated by OrthoFinder. The alignments were filtered using TRIMAL (v1.4. rev22) [26] to trim alignment results and connect them end-to-end to form a super-long sequence for constructing the evolutionary tree. IQ-TREE (v2.1.2) [27] with parameters “-m MFP -bb 1000” was used to construct the maximum likelihood (ML) phylogenetic tree with the best model (JTT + F + R5) estimated using ModelFinder [28] and 1000 bootstrap replicates. MCMCtree in the PAML package (v4.10.5) [29] was used to estimate the divergence times between these species. Five calibration time points based on fossil records from the TimeTree [30] database were used: (a) stem *Orussus. abietinus*, 211–289 million years ago (Mya); (b) stem Aculeata, 160–224 Mya; (c) stem Ichneumonidae, 151–218 Mya; (d) stem Chalcidoidea, 105–159 Mya; and (e) stem Apoidea, 93–132 Mya. FigTree (v1.4.4) (http://tree.bio.ed.ac.uk/software/figtree/, accessed on 10 March 2026) was used to visualize the tree structure.

### 2.2. Analysis of Gene Family Expansion and Contraction

Gene family expansion and contraction were analyzed using CAFE (v5.0) [31]. Gene count files of individual gene families generated by Orthofinder and phylogenetic trees with clade lengths were used as the inputs. The expanded and contracted gene families of *Er. hayati* were used to perform GO enrichment analysis using GOATOOLS [32] and KEGG pathway enrichment analysis via the Omicshare online platform. GO enrichment results were visualized using REVIGO [33].

### 2.3. Positive Selection Gene Analysis

Five species from Aphelinidae, nine species from Chalcidoidea, as well as *Cotesia chilonis* and *Belonocnema treatae*, were employed to identify the positively selected genes. The phylogenetic tree was constructed according to the methods described above. OrthoFinder was used to identify single-copy homologous proteins from these species. MAFFT was used to align protein sequences in each orthologous group, and PAL2NAL (v14) [34] was used to obtain codon-aligned sequences based on the corresponding coding sequences (CDS). We used the branch-site model of CodeML to calculate the dN/dS ratio for each gene tree.

Specifically, we labeled the target evolutionary branches as foreground and the other branches as background. Then, the lnl values for both the original and alternative hypotheses were calculated. The chi2 statistic was used for the chi-square test after the lnl value difference (2∆l = 2 × (l_1_ − l_0_)) was calculated. We chose *p* = 0.05 as the critical significance *p*-value after false discovery rate (FDR) correction to identify positively selected orthogroups.

### 2.4. Analysis of Chromosome Synteny

Chromosome synteny between *Er. hayati*, *En. formosa* and *Nasonia vitripennis* was analyzed using MCSCANX [35] with default parameters. Orthologous protein sequences were determined using Blastp software (v2.14.0) (E-value < 1 × 10^−10^). MCSCANX results were visualized using Cicros (v0.69.9) [36].

Intraspecific collinearity analysis was performed using WGDI (v0.6.5) [37]. The protein sequences of the three species were self-aligned using Blastp. The collinearity function was employed to preliminarily identify collinear regions using the parameters “multiple = 2, evalue = 1 × 10^−5^, score = 100, position = order”. The ks function was used to invoke MUSCLE to calculate the Ks value for each gene pair. The blockinfo function extracts collinear block information with the parameter “ks_col = ks_NG86”. A correspondence function was applied to filter the block data using the parameters “block_length = 5, tandem_ratio = 0.5”. Finally, the blocks function computed the Ks values for each collinear block and output the collinearity results.

### 2.5. Genome Size and LTR Insertion Analysis

The genome sizes of all species included in the phylogenetic tree were collected, and ancestral state reconstruction was performed using the R package Phytools (v1.2-0) in conjunction with tree topology. Transposable elements (TEs) were identified and extracted from the genomes of *Er. hayati*, *En. formosa*, *Copidosoma floridanum*, *Trichogramma pretiosum*, *Pteromalus puparum* and 12 other species using EDTA (v2.0.1) [38]. For each intact LTR retrotransposon, the sequences from the two terminal LTRs were extracted and aligned using MAFFT (v7.471). Subsequently, the insertion time of LTR retrotransposons was estimated based on the alignment results of the terminal LTR sequences using the R package APE (v5.6-2) [39].

## 3. Results

### 3.1. Phylogenetic Analysis and Dynamics of Gene Families

In total, 92.5% of the 745,029 protein-coding genes from the 40 Hymenoptera species were classified into 42,924 orthogroups using the OrthoFinder (v2.5.4) software. Among these orthogroups, 1264 were present in all species and 156 were single-copy orthogroups (Supplementary Table S3). In *Er. hayati*, 22,226 genes were assigned to 9789 orthogroups, including 962 species-specific orthogroups that contained a total of 6171 species-specific genes, which is significantly higher than that in other species (Supplementary Table S4, Supplementary Figure S1). In addition, orthogroups were extracted from *Er. hayati* and four other Aphelinidae species (*En. formosa*, *Encarsia sophia*, *Aphelinus certus* and *Aphelinus atriplicis*). A total of 5816 orthogroups were present in all five species, whereas *Er. hayati* had 1333 species-specific orthogroups (Supplementary Figure S2).

**Figure 1:**
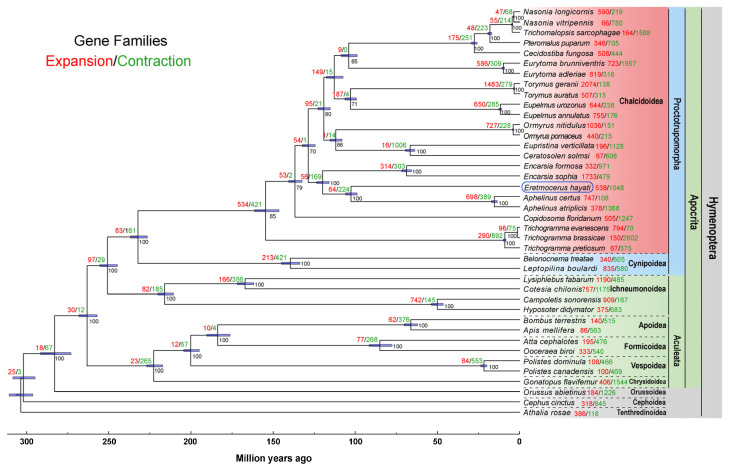
Phylogenetic analysis and gene family expansion/contraction analysis. The maximum likelihood evolutionary tree of *Eretmocerus hayati* (blue rectangular marker) and 39 other species of Hymenoptera was constructed using IQ-TREE with 156 connected single-copy orthologous protein sequences and 1000 bootstrap replicates. *Athalia rosae* was used as the outgroup. Different taxa are marked with differently colored regions. The light blue columns at the branching nodes represent the evolutionary divergence times predicted by MCMCtree with 95% confidence. The numbers of expansion and contraction gene families within each internode and species, calculated using the CAFE (v5.0) software, are shown in red and green, respectively. The numbers following branch nodes represent the Bootstrap values.

The phylogenetic analysis indicated that the Aphelinidae species *Er. hayati*, *A. certus* and *A. atriplicis* clustered together, with *En. formosa* and *En. sophia* forming a sister group to this clade (Figure 1). Furthermore, species in Aphelinidae were clustered into a sister group to other species in Chalcidoidea, excluding Trichogrammatidae and Encyrtidae. This topology is consistent with a previous analysis of Chalcidoidea in Hymenoptera [40,41]. The MCMCTree results indicated that the Aphelinidae lineage commenced its diversification approximately 119.9 Mya, while the speciation time of *Eretmocerus* and *Aphelinus* was estimated to be 102.6 Mya (Figure 1).

At the ancestral divergence point of Aphelinidae, a total of 56 expanded gene families and 169 contracted gene families were identified. Within *Er. hayati*, 538 expanded and 1048 contracted gene families were identified, whereas *En. formosa* displayed 332 expanded and 971 contracted gene families (Figure 1). Compared to *En. formosa*, 480 expanded gene families were exclusive to *Er. hayati,* 58 expanded gene families were in both species; 796 contracted gene families were exclusive to *Er. Hayati*, and 252 contracted gene families were in both species (Supplementary Figure S3).

### 3.2. Gene Family Expansion and Contraction Analysis

**Figure 2:**
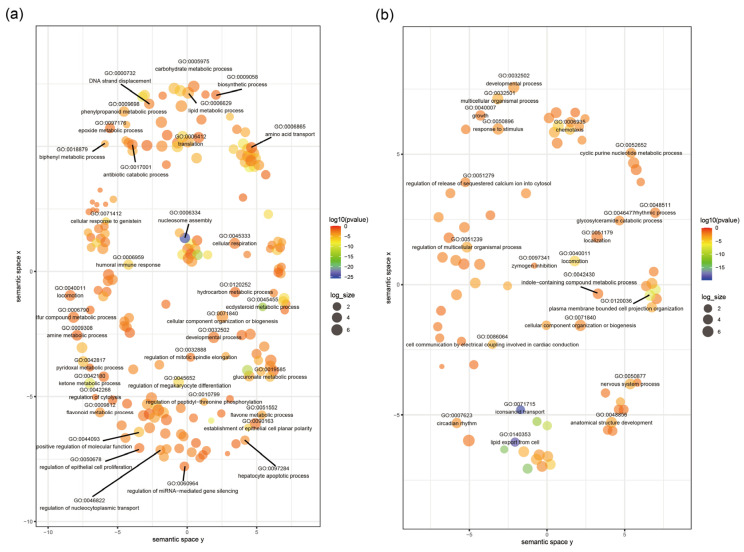
Gene Ontology (GO) enrichment of expanded (**a**) and contracted (**b**) orthogroups of *Eretmocerus hayati*. Different point colors and sizes represent different log_10_-transformed *p*-values and the number of annotations for GO term identity in the EBI GOA database, respectively. According to the results of REVIGO analysis, only GO terms with a dispensability score < 0.15 (i.e., the most representative terms in each cluster) are labeled to ensure clarity. Dispensability is defined as the semantic similarity threshold at which a term is merged into a cluster representative.

GO and REVIGO analyses revealed that the expanded orthogroups in *Er. hayati* were enriched in DNA replication regulation (e.g., protein-DNA complex subunit organization, DNA strand displacement, chromosome organization and nucleosome assembly), metabolism-related pathways (e.g., glucuronate metabolic process, peptidoglycan metabolic process, carbohydrate metabolic process, lipid metabolic process and amino acid transport), and immune response (e.g., response to stimulus, humoral immune response, detoxification and positive regulation of melanization defense response) (Figure 2a, Supplementary Figure S4, Supplementary Table S5). In contrast, contracted orthogroups were significantly enriched in eicosanoid transport, lipid export from the cell, cyclic purine nucleotide metabolic processes, and circadian rhythm (Figure 2b, Supplementary Table S6).

The expanded and contracted gene families in *Er. hayati* were enriched in several KEGG pathways. Expanded orthogroups were significantly enriched in pathways including carbohydrate metabolism (e.g., Galactose metabolism, pentose and glucuronate interconversions), lipid metabolism (e.g., steroid hormone biosynthesis and fatty acid biosynthesis), signal transduction (e.g., AMPK signaling pathway and Calcium signaling pathway), endocrine system (e.g., Renin-angiotensin system and PPAR signaling pathway), immune diseases and systems (e.g., neutrophil extracellular trap formation and hematopoietic cell lineage), and xenobiotic biodegradation and metabolism (e.g., metabolism of xenobiotics by cytochrome P450 and drug metabolism by cytochrome P450). Contracted orthogroups were mainly enriched in pathways including the endocrine system, viral infectious diseases, digestive system, nervous system, and signal transduction pathways.

### 3.3. Positive Selection Genes

To identify genes under positive selection in *Er. hayati*, we reconstructed a phylogenetic tree comprising five species from the Aphelinidae, two species from the Pteromalidae, as well as one representative species from each of the other seven families within Chalcidoidea included in Figure 1. *Cotesia chilonis* (Ichneumonoidea) and *Belonocnema treatae* (Cynipoidea) were selected as outgroups. In these species, 1060 single-copy orthologs were identified for subsequent positive selection analysis.

**Figure 3:**
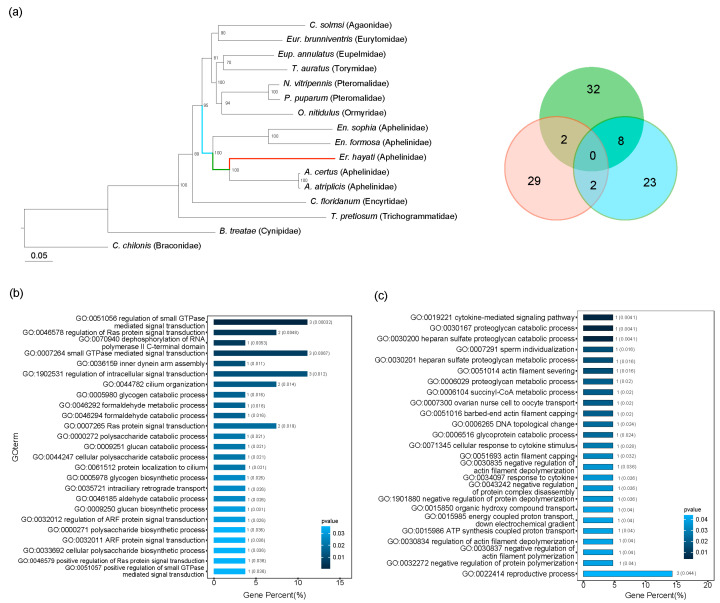
Positive selection gene analysis. (**a**) Phylogeny of 16 hymenopteran species and the distribution of positively selected genes across the evolutionary branch of *Eretmocerus hayati* and its ancestral branches. The maximum likelihood (ML) tree was reconstructed based on the single-copy orthologs. The tree includes five species from the family Aphelinidae, along with representative species from each of the nine families within Chalcidoidea included in Figure 1. *Cotesia chilonis* (Ichneumonoidea) and *Belonocnema treatae* (Cynipoidea) were used as outgroups. The numbers following branch nodes represent the Bootstrap values. The colors of the areas of the Venn diagram represent different branches of the phylogenetic tree. (**b**,**c**) The top 25 enriched GO terms of positively selected genes of *Er. hayati* and the Aphelinidae ancestor branch node, respectively. The vertical axis represents the GO number and the specific description. The number of genes and *p*-values in each GO term are marked to the right of the column.

We identified 33 genes under positive selection in *Er. hayati*, 29 of which were species-specific. In addition, 33 positively selected genes were identified in the ancestral branch of Aphelinidae and 42 were identified in the ancestral branch of *Eretmocerus* and *Aphelinus* (Figure 3a). Among these, eight genes were positively selected in both ancestral branches, including Cleft lip and palate transmembrane protein 1 (CLPTM1), DNA Topoisomerase 1 (TOP1) (resolving torsional stress in DNA during replication, transcription, and chromatin condensation) [42,43], Importin 13 (IPO13) (nuclear transport receptor involved in import and export of cargoes, affecting neurotransmitter release at synapses) [44,45], C2H2-type zinc finger protein (BCL11A) (regulating neural development, temporal patterning of neural stem cells, and locomotor behavior) [46,47], RNA-binding protein MEX-3 (MEX3C) (participates in immune responses within hemocytes) [48], DEAH-Box Helicase 34 (DHX34) (playing roles in RNA helicase activity for mRNA surveillance and splicing) [49,50], chromodomain helicase DNA binding protein 7 (CHD7) (encoding a protein that contains several helicase family domains and is involved in nucleosome binding and remodeling activities) [51,52] and Hormone receptor 39 (Hr39) (involved in reproduction, vitellogenin synthesis, developmental regulation, and ecdysone signaling pathways) [53,54,55]. These genes were involved in physiological activities such as DNA replication and recombination, signal transduction, immune regulation, and reproductive control. When focusing on the genes under positive selection both in the *Eretmocerus* branch and its most recent ancestral branch, leucine-rich repeat scaffold protein (SHOC2) and S-formylglutathione hydrolase (ESD) were identified, which participate in the positive regulation of antiviral processes [56] and degradation of toxins [57,58], respectively.

The 29 specific positively selected genes in *Er. hayati* were enriched in GO terms primarily involved in signaling and metabolic processes, such as GTPase-mediated signal transduction, regulation of Ras protein signal transduction, regulation of intracellular signal transduction, glucan catabolic process, polysaccharide catabolic process, and cellular polysaccharide catabolic process (Figure 3b). In addition, the 33 positively selected genes in the Aphelinidae ancestral branch were enriched in GO terms such as proteoglycan catabolic process, DNA topological change, cellular response to cytokine stimulus, sperm individualization, and ovarian nurse cell-to-oocyte transport (Figure 3c).

### 3.4. Complex Chromosomal Collinearity

**Figure 4:**
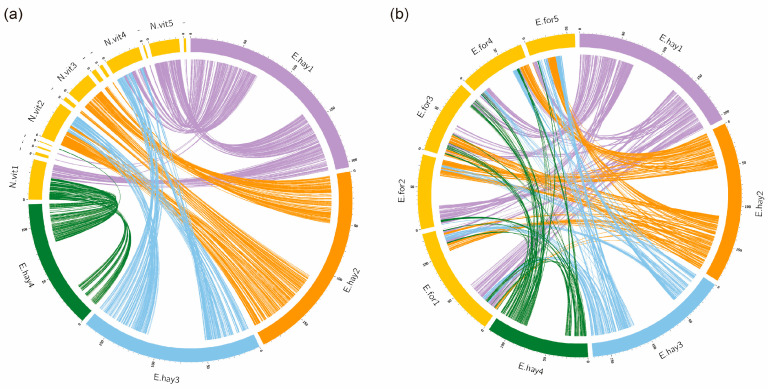
Chromosomal synteny analysis. (**a**) Chromosome synteny between *Er. hayati* and *N. vitripennis.* (**b**) Chromosome synteny between *Er. hayati* and *En. formosa*. The colors of the connecting lines depend on the *Er. hayati* chromosome on which the gene is located. Owing to the limited space of the diagrams, the species names are abbreviated, followed by the figure of the chromosome number (for example, E.hay1 represents chromosome 1 of *Er. hayati*). The minus symbol “-” represents the episomal genome sequence that could not be located on chromosomes.

To characterize chromosomal collinearity in *Er. hayati*, we performed comparative analyses with *En. formosa* (a closely related species) and *N. vitripennis* (a distantly related species). A total of 383 synteny blocks (each containing at least five orthologous genes) were identified between *Er. hayati* and *En. Formosa.* Of these, 227 blocks (59.3%) contained more than 10 orthologous genes, with an average of 20.2 genes per block. When *Er. hayati* was compared with *N. vitripennis*, 378 synteny blocks were identified, of which 230 (60.8%) contained more than 10 orthologous genes, with an average gene number of 21.3 per block (Supplementary Figure S5). Although the average number of genes per synteny block was similar in both comparisons, *Er. hayati* showed better synteny with *N. vitripennis* than with the closely related species *En. formosa*, as shown by the distribution of the homologous blocks across chromosomes (Figure 4). The complex chromosomal collinearity between *Er. hayati* and *En. formosa* may indicate extensive chromosomal fission and fusion events in these two species.

**Figure 5:**
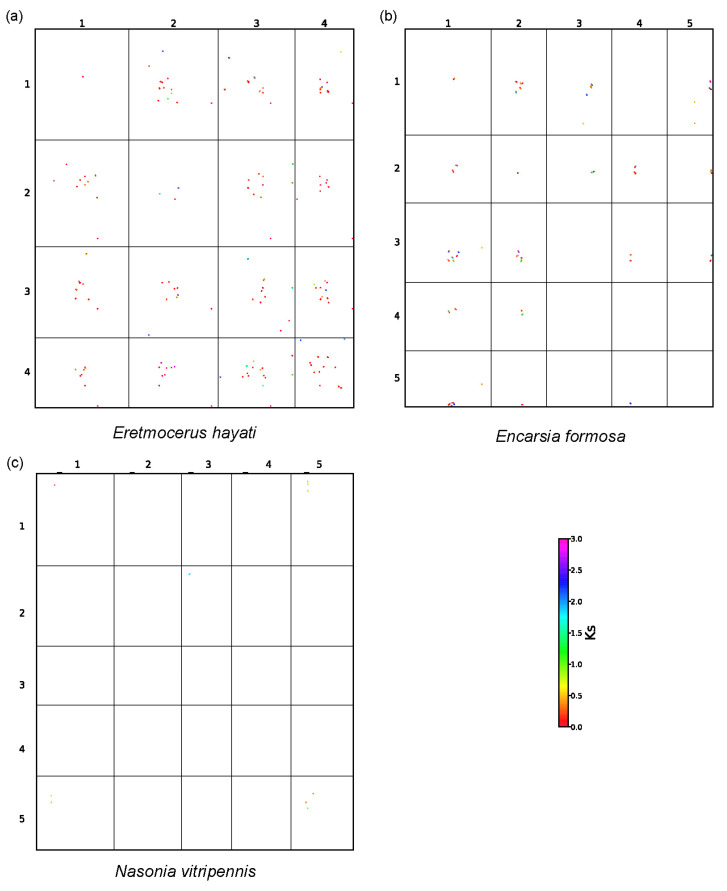
Analysis of chromosome collinearity in (**a**) *Eretmocerus hayati*, (**b**) *Encarsia formosa* and (**c**) *Nasonia vitripennis*. The coordinates are the chromosome numbers. The color of the points represents the Ks value of the collinearity regions.

The intraspecific collinearity analysis revealed clear differences among the three species. *N. vitripennis* showed almost no collinearity within its own chromosomes, with only a few syntenic regions detected (for example, between chromosomes 1 and 5, Figure 5c). In contrast, both *En. formosa* and *Er. hayati* exhibited a greater number of intraspecific collinear regions, and *Er. hayati* had notably more such regions than *En. formosa* (Figure 5a,b). The high level of intraspecific synteny in *Er. hayati* may partially explain the complex chromosomal collinearity between *Er. hayati* and *En. formosa*.

### 3.5. The Larger Genome Size and the Recent Outbreak of LTR Sequence Insertion

**Figure 6:**
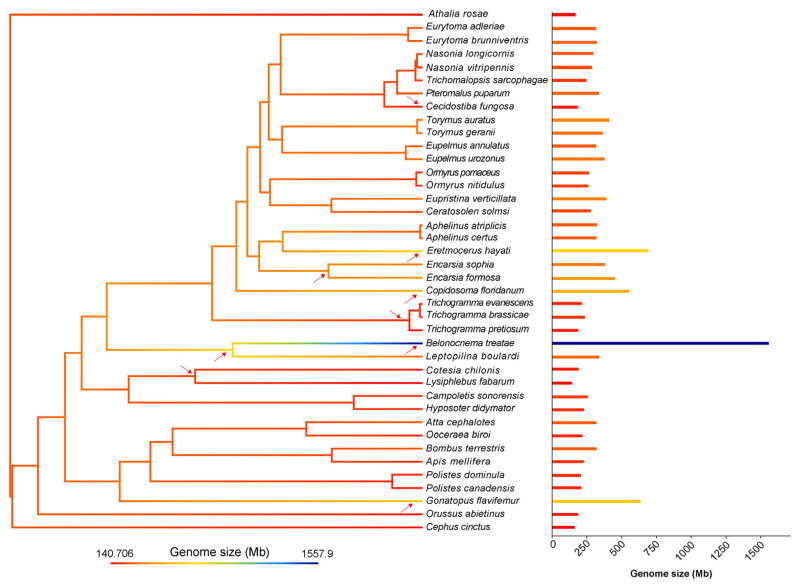
Ancestral state reconstruction of genome size across selected Hymenoptera. The phylogenetic tree (**left**) illustrates the ancestral state reconstruction of genome size, with arrows indicating nodes or branches where notable changes in genome size occurred. The bar chart (**right**) displays the genome size of each species included in the analysis.

Among the species included in the phylogenetic tree, *Belonocnema treatae* possessed the largest genome, with an estimated size of approximately 1.5 Gb, which was substantially greater than that of any other species examined in this study. Genome sizes of the remaining species ranged from 140 Mb to 692 Mb (Figure 6). Ancestral state reconstruction identified several key nodes within these hymenopteran lineages where significant genome size changes occurred. Basal lineages, such as *Athalia rosae*, *Cephus cinctus*, and the parasitic wood wasp *Orussus abietinus*, had relatively small genomes. Genome size reduction was observed in the braconid clade (containing *Cotesia chilonis* and *Lysiphlebus fabarum*) and in the *Trichogramma* clade (comprising *Trichogramma pretiosum*, *Trichogramma evanescens*, and *Trichogramma brassicae*). In contrast, genome size expansion occurred in the dryinid clade (including *Gonatopus flavifemur*) and the cynipid clade containing *B. treatae*. Furthermore, the *Encarsia* clade and the *Eretmocerus* clade within Aphelinidae, as well as *C. floridanum*, showed a notable increase in genome size (Figure 6).

**Figure 7:**
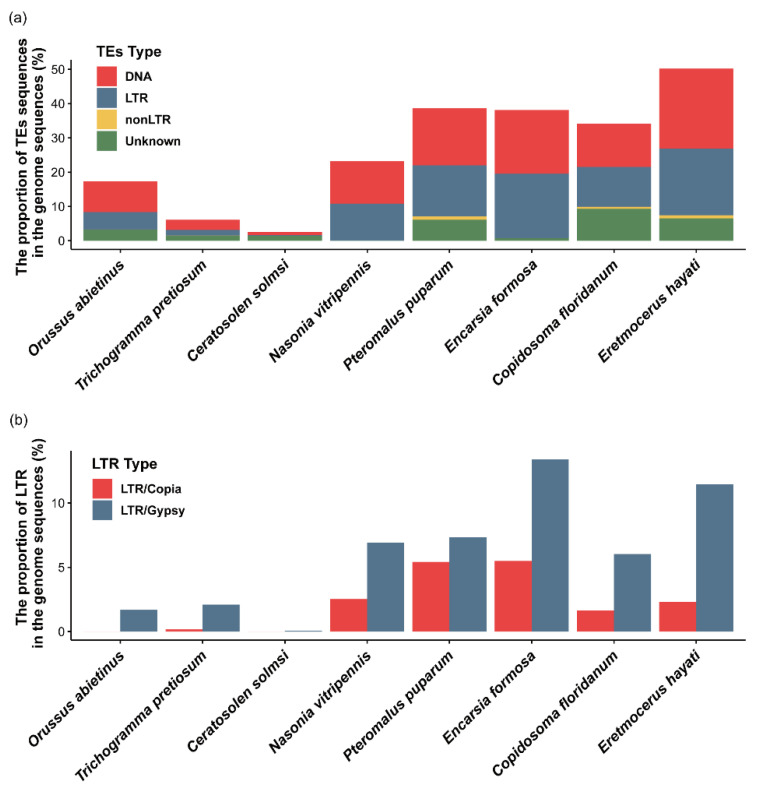
Proportion of repeated sequences in the genomes of different species of Hymenoptera. (**a**) Visualization of the proportion of various transposon sequences in the eight species. Species are ordered by increasing genome size from left to right. (**b**) Proportion of the two LTR sequences in the genome.

To investigate whether transposable elements (TEs) contributed to the large genome size, as TEs are known major drivers of genome expansion, eight representative species were selected on the basis of genome size and phylogenetic relationships for TE analysis. Results showed that TEs accounted for 50.19% of the *Er. hayati* genome and 53.27% of the *G. flavifemur* genome. The genome of *B. treatae*, which has the largest genome size, had the highest TE content, at 60.61%. Examination of the relative proportions of different TE categories indicated divergence in the dominant TE types among species (Supplementary Table S7, Figure 7a). DNA transposons were the dominant TE type in *B. treatae* and *G. flavifemur*, making up 71.87% and 75.35% of all TE sequences, respectively. In contrast, DNA transposons accounted for only 46.50% and 48.74% of the total TE content in *Er. hayati* and *En. formosa*, respectively. Among all analyzed species, *Er. hayati* exhibited the highest genomic proportion of LTR retrotransposons (19.44%), followed by *En. formosa* (18.76%) (Supplementary Table S7).

A comparison of the two major LTR retrotransposon categories across the eight hymenopteran species showed that gypsy elements were generally more abundant than copia elements (Figure 7b). Analysis of TE category proportions across species revealed a clear correlation between genome size and TE content in hymenopterans, along with distinct patterns of TE composition among different hymenopteran lineages.

**Figure 8:**
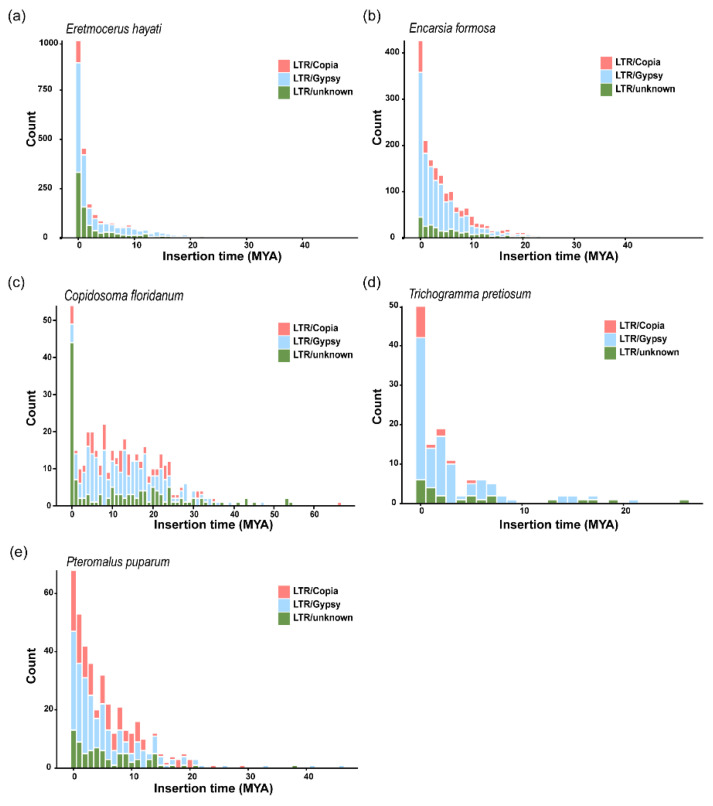
LTR sequence insertion time of five Hymenoptera species. (**a**–**e**) Number of LTR sequences and insertion time predicted for each species. The vertical axis shows the number of insertions, and the horizontal axis shows the estimated insertion time points. Different colors in the bar chart represent different LTR sequence types.

Further analysis was performed to estimate the insertion times of LTR retrotransposons in the five hymenopteran species. The results showed that, except for *C. floridanum*, the majority of LTR insertions in the other four species occurred within the past 20 million years. The genomes of *En. formosa* and *Er. hayati* contained substantially more LTR sequences than those of the other three species. A burst of LTR retrotransposon activity was observed within the last 1 million years in *C. floridanum*, *Er. hayati* and *En. formosa* (Figure 8). In *Er. hayati* and *En. formosa*, this recent proliferation was driven mainly by gypsy elements, whereas *C. floridanum* showed a marked increase in unclassified LTR sequences. These estimated insertion times of LTR retrotransposons suggest that the recent bursts of LTR activity are likely a major contributor to the significant genome size expansion observed in the evolutionary lineages of *Er. hayati* and *En. formosa* within Hymenoptera.

## 4. Discussion

Chalcidoidea is a highly diverse superfamily within Hymenoptera that comprises a large number of species. Analyses of morphological characteristics and molecular data indicate that Chalcidoidea might have diverged from the common ancestor shared with other parasitic Hymenoptera approximately 150 million years ago [40]. Our results show that the family Aphelinidae split from other hymenopteran lineages around 120 million years ago and formed an independent clade. Notably, *Eretmocerus*, *Aphelinus* and *Encarsia* represent early-diverging lineages within Aphelinidae (Figure 1). This finding corroborates the established view that *Eretmocerus* is distinct from other Aphelinidae genera [59]. Nevertheless, the current divergence time estimates for Aphelinidae and *Eretmocerus* are preliminary, as our phylogeny included only five species of Aphelinidae and nine families of Chalcidoidea. With the rapid growth of genomic resources for Hymenoptera, future studies with broader taxon sampling are expected to yield more accurate divergence time estimates.

Species in the superfamily Chalcidoidea display diverse parasitic strategies, ranging from ectoparasitism to endoparasitism or intermediate forms between these two methods [1]. The parasitic behavior of *Er. hayati* represents an intermediate form between endoparasitism and ectoparasitism: females deposit eggs in the space between the host nymph and the plant surface rather than injecting them directly into the host body [60]. Upon hatching, the first-instar larva penetrates the host abdomen but remains external; it only enters the host to complete development once the nymph reaches its final stage [61,62]. During the first and second instars, the larva is enclosed in a host-derived capsule and does not contact host tissues directly until the third instar. Parasitic wasps typically inject venom to regulate the host’s immune response, thereby facilitating egg and larval development inside or on the host body. However, the oviposition strategy of *Er. hayati* may limit direct and effective venom delivery into the host, weakening its ability to inhibit host immunity. We therefore hypothesize that *Er. hayati* has evolved enhanced defense mechanisms to counteract the host immune response. Thus, gene families involved in humoral immunity have undergone adaptive expansion in its genome (Supplementary Figure S4).

Additionally, we found that gene families related to energy metabolism in the genome of *Er. hayati* have undergone significant expansion. This finding is closely linked to the physiological characteristics imposed by the miniaturization of parasitic wasps. Such miniaturization of parasitic wasps notably increases their metabolic requirements. This increased metabolic demand, often achieved through higher energy expenditure, drives them to prioritize high-energy food resources [63]. Moreover, smaller parasitic wasps generally exhibit superior flight capabilities. These characteristics enable them to adapt more effectively to foraging or host-seeking in intricate or restricted ecological settings [2,64,65]. As a micro-sized species with a body size of less than 3 mm, *Er. hayati* demonstrates remarkable flight and host-searching capabilities that necessitate a high-energy metabolic capacity [66]. The observed expansion of energy metabolism gene families in the *Er. hayati* genome (Figure 2a, Supplementary Figure S4) likely represents a genomic adaptation to support its high-energy lifestyle requirements.

This study revealed that genes involved in nucleosome binding and remodeling activities have evolved rapidly under continuous positive selection along the ancestral branch of Aphelinidae and *Eretmocerus* (Figure 3a,b), which may underlie the high level of intraspecific chromosomal collinearity observed between *Er. hayati* and *En. Formosa* (Figure 5). Chromosomal variations, such as duplications and translocations, can enhance the genomic diversity of insects [67,68], thereby improving their adaptability to environmental changes [69]. The complex chromosomal structural variations and numerous species-specific gene families found in *Er. hayati* likely contribute to its high genomic complexity and structural redundancy (Figure 5, Supplementary Figure S1). This structural redundancy may not only improve its adaptability to environmental changes but also reduce the risk of inbreeding depression, thereby enabling rapid population expansion from a small founding group. These features provide a genetic basis for the successful large-scale rearing of this species. Furthermore, Chalcidoidea exhibits extensive chromosomal diversity, with species in Aphelinidae possessing 3 to 11 chromosomes [70]. The complex chromosomal collinearity patterns in *Er. hayati* revealed in this study offer important insights into the evolutionary mechanisms underlying chromosome number diversity in this family, e.g., through repeated chromosomal fission and fusion events.

Existing genomic data on parasitic wasps reveal substantial variation in genome size across species, ranging from 180 Mb to 878 Mb [12,13]. Even within the same family, genome size can differ by several fold [2]. *Er. hayati*, *En. formosa*, and *C. floridanum* possess relatively larger genomes compared with other Chalcidoidea species; however, the mechanisms underlying their genomic expansion appear to be distinct. *Er. hayati* exhibited the highest number of recent LTR-Gypsy sequence insertions (Figure 8a), whereas most LTR sequences in *C. floridanum* belonged to unknown types (Figure 8c). Studies suggest that LTR insertions in parasitic wasp genomes typically do not affect exon length but instead increase intron length [14]. Excessive genomic expansion may compromise genome stability [71,72], and some species have been shown to undergo Piwi gene expansion to suppress the effects of transposable elements (TEs) [14]. However, not all species with TE insertions exhibit Piwi gene expansion [73,74]. Therefore, the mechanisms contributing to genomic stability in *Er. hayati* require further investigation.

## Data Availability

The original data presented in the study are openly available in Figure Share at 10.6084/m9.figshare.22709485 and 10.6084/m9.figshare.22709494.

## Supplementary Materials

The following supporting information can be downloaded at: https://www.mdpi.com/article/10.3390/insects17040369/s1, Figure S1: Genetic classification of 40 species of Hymenoptera classified by Orthofinder; Figure S2: Venn diagram of orthogroups from five Aphelinidae species; Figure S3: Venn diagram of the expanded and contracted gene families in *Er. hayati* and *En. formosa*; Figure S4: REVIGO treeplot of biological process GO enrichment results of expanded gene families in *Er. hayati*; Figure S5: Number of genes in each syntenic block between two species and *Er. hayati*; Table S1: Transcriptome sample name abbreviations; Table S2: Data sources of protein-coding sequences used in this study; Table S3: Summary of all orthogroups identified by OrthoFinder; Table S4: Results of gene classification in *Er. hayati*; Table S5: GO enrichment of expanded genes in *Er. hayati*; Table S6: GO enrichment of contracted genes in *Er. hayati*; Table S7: Proportions of various transposons in the genome sequences of some hymenopteran species.
