# Supplementary material for: Genomic Expansion and Adaptation in a Parasitoid Wasp Eretmocerus hayati (Hymenoptera: Aphelinidae)
Source: Insects. 2026 Mar 31;17(4):369. doi: 10.3390/insects17040369 (PMC13116730; doi:10.3390/insects17040369)
Supplement: Supplementary file 1 [file insects-17-00369-s001.zip › insects-4166988-supplementary.pdf]

Supplement Figures

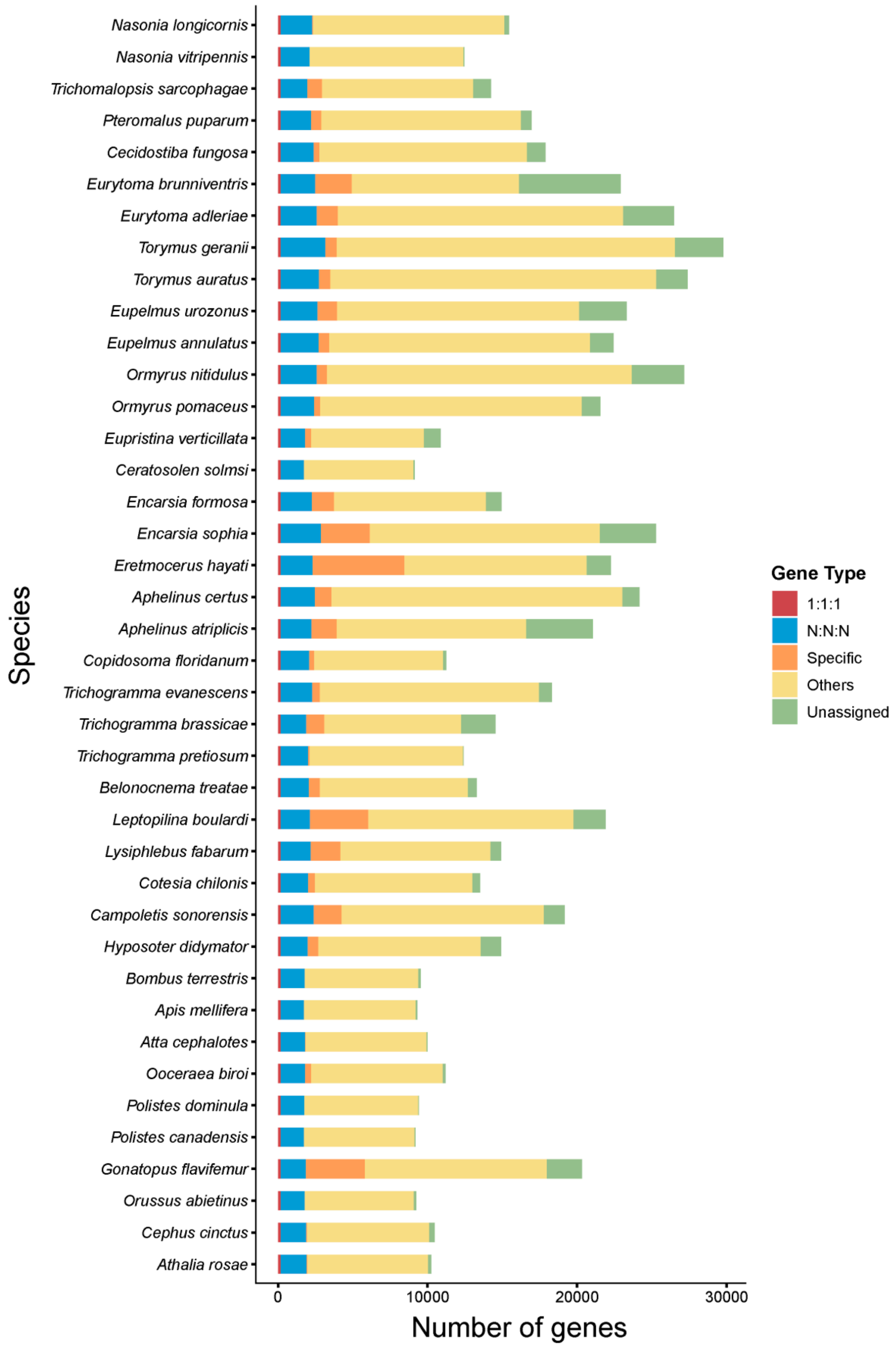

Supplementary Figure S1. Genetic classification of 40 species of Hymenoptera classified by Orthofinder. Unassigned represents genes that have not been successfully clustered. 1:1:1 represents single copy genes in all species. N:N:N represents genes present in all species, but not a single copy.

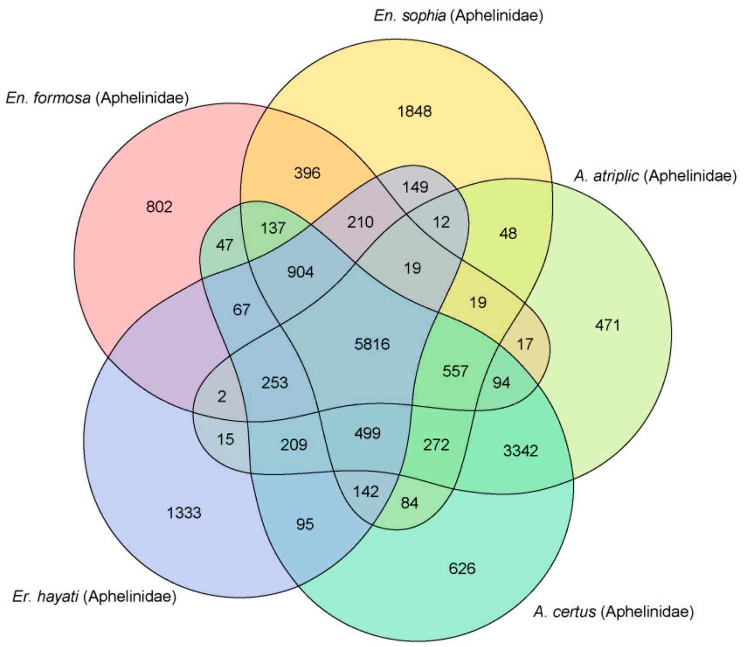

Supplementary Figure S2. Venn diagram of orthogroups from five Aphelinidae species. Numbers in the same color regions represent orthogroups including genes from the same species, and numbers in the overlapping regions represent orthogroups with genes from different species.

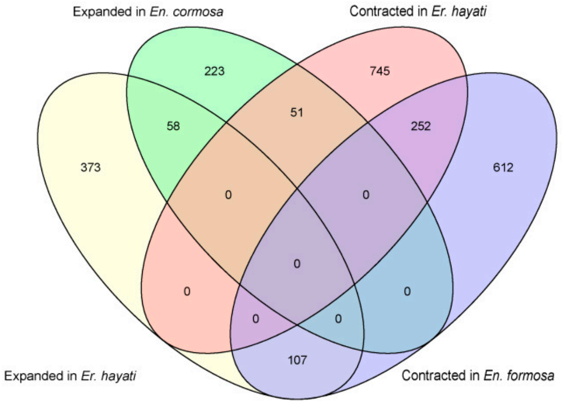

Supplementary Figure S3. Venn diagram of expanded and contracted gene families in *Er. hayati* and *En. formosa*. Numbers represent orthogroups that are expanded or contracted in *Er. hayati* and *En. formosa*.

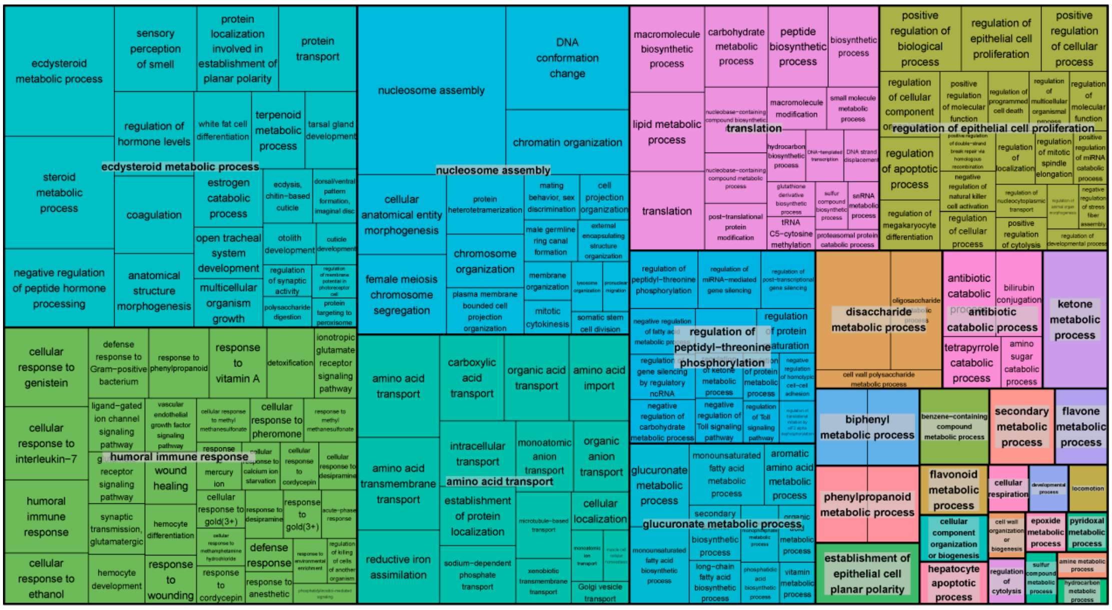

Supplementary Figure S4. REVIGO treeplot of biological process GO enrichment results of expanded gene families in *Er. hayati*. Representative GO entry names are marked with semi-transparent rectangles.

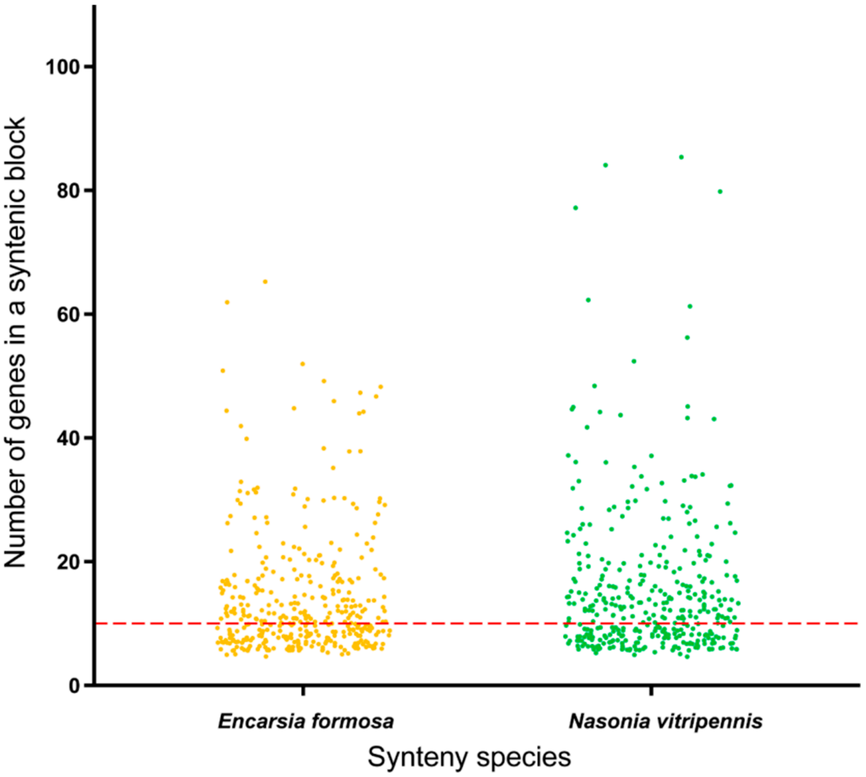

Supplementary Figure S5. Number of genes in each syntenic block between two species and *Er. hayati*. Each point represents a collinear block, and the red dotted line represents the number of genes in the block is ten.

Supplement Tables

Supplement Table S1. Transcriptome sample name abbreviation

Abbreviation	Description
E	Eggs laid within 12 hours
EL	Early larva (first and second instar)
LL	Later larva (third and fourth instar)
FA	Whole body of female adult within 12 hours of emergence
MA	Whole body of male adult within 12 hours of emergence
FH	Heads of female adult within 12 hours of emergence
MH	Heads of male adult within 12 hours of emergence
FA_heat	Whole body of female adult treated at 35 degree centigrade for 48 hours

Supplementary Table S2. The data source of protein-coding sequences used in this study.

Name of speciess	Family	Data source
Aphelinus atriplicis	Aphelinus	https://insect-genome.com/genome/IBG_03234
Aphelinus certus	Aphelinus	https://insect-genome.com/genome/IBG_03235
Apis mellifera	Apidae	https://insect-genome.com/genome/IBG_00083
Athalia rosae	Athaliidae	RefSeq assembly accession： GCF_917208135.1
Atta cephalotes	Formicidae	RefSeq assembly accession： GCF_917208135.1
Belonocnema treatae	Cynipidae	https://insect-genome.com/genome/IBG_00109
Bombus terrestris	Apidae	RefSeq assembly accession： GCF_910591885.1
Campoletis sonorensis	Ichneumonidae	https://insect-genome.com/genome/IBG_00159
Cecidostiba fungosa	Pteromalidae	https://insect-genome.com/genome/IBG_00165
Cephus cinctus	Cephidae	RefSeq assembly accession： GCF_000341935.2
Ceratosolen solmsi	Agaonidae	RefSeq assembly accession： GCF_000503995.1
Copidosoma floridanum	Encyrtidae	RefSeq assembly accession： GCF_000648655.2
Cotesia chilonis	Braconidae	https://insect-genome.com/genome/IBG_00206
Encarsia formosa	Aphelinidae	Shenyang Agricultural University unpublished
Encarsia sophia	Aphelinidae	https://insect-genome.com/genome/IBG_02793
Eretmocerus hayati	Aphelinidae	This study
Eupelmus annulatus	Eupelmidae	https://insect-genome.com/genome/IBG_00379
Eupelmus urozonus	Eupelmidae	https://insect-genome.com/genome/IBG_00380
Eupristina verticillata	Agaonidae	https://insect-genome.com/genome/IBG_00382
Eurytoma adleriae	Eurytomidae	https://insect-genome.com/genome/IBG_00384
Eurytoma brunniventris	Eurytomidae	https://insect-genome.com/genome/IBG_00385
Gonatopus flavifemur	Dryinidae	https://insect-genome.com/genome/IBG_00409
Hyposoter didymator	Ichneumonidae	https://insect-genome.com/genome/IBG_00464
Leptopilina boulardi	Cynipidae	RefSeq assembly accession： GCF_019393585.1
Lysiphlebus fabarum	Braconidae	https://insect-genome.com/genome/IBG_00524
Nasonia longicornis	Pteromalidae	https://insect-genome.com/genome/IBG_00563
Nasonia vitripennis	Pteromalidae	RefSeq assembly accession： GCF_009193385.2
Ooceraea biroi	Formicidae	RefSeq assembly accession： GCF_003672135.1
Ormyrus nitidulus	Ormyridae	https://insect-genome.com/genome/IBG_00594
Ormyrus pomaceus	Ormyridae	https://insect-genome.com/genome/IBG_00595
Orussus abietinus	Orussidae	RefSeq assembly accession： GCF_000612105.2
Polistes canadensis	Vespidae	RefSeq assembly accession： GCF_001313835.1
Polistes dominula	Vespidae	RefSeq assembly accession： GCF_001465965.1
Pteromalus puparum	Pteromalidae	https://insect-genome.com/genome/IBG_00672
Torymus auratus	Torymidae	https://insect-genome.com/genome/IBG_00759
Torymus geranii	Torymidae	https://insect-genome.com/genome/IBG_00760
Trichogramma brassicae	Trichogrammatidae	https://insect-genome.com/genome/IBG_00770
Trichogramma evanescens	Trichogrammatidae	https://insect-genome.com/genome/IBG_00771
Trichogramma pretiosum	Trichogrammatidae	RefSeq assembly accession： GCF_000599845.2
Trichomalopsis sarcophagae	Pteromalidae	https://insect-genome.com/genome/IBG_00773

Supplementary Table S3. Summary of all orthogroups identified by OrthoFinder.

Category	Value
Number of species	40
Number of genes	745,029
Number of genes in orthogroups	688,780
Number of unassigned genes	56,249
Percentage of genes in orthogroups	92.5%
Percentage of unassigned genes	7.5%
Number of orthogroups	42,924
Number of species-specific orthogroups	10,679
Number of genes in species-specific orthogroups	51,871
Percentage of genes in species-specific orthogroups	7
Mean orthogroup size	16
Median orthogroup size	4.0
G50 (assigned genes)	41
G50 (all genes)	40
O50 (assigned genes)	4,606
O50 (all genes)	5,296
Number of orthogroups with all species present	1,264
Number of single-copy orthogroups	156

*G50 and O50: When we arrange the orthogroups in descending order according to the gene number and add them until the total number of genes in orthogroups is greater than 50% of the total number of genes, the number of orthogroups required is O50 and the number of genes in that orthogroup is G50.

Supplementary Table S4. Results of genes classification in *Er. hayati*.

Category	***Eretmocerus hayati***
Number of genes	23,916
Number of genes in orthogroups	22,226
Number of unassigned genes	1,650
Percentage of genes in orthogroups	93.1%
Percentage of unassigned genes	6.9%
Number of orthogroups containing species	9,789
Percentage of orthogroups containing species	22.8%
Number of species-specific orthogroups	962
Number of genes in species-specific orthogroups	6,171
Percentage of genes in species-specific orthogroups	25.8%

Supplementary Table S5. GO enrichment of expanded genes in *Er. hayati*, p_fdr < 0.05**.**

GO	Description	Study	Pop	p_fdr
GO:0006334	nucleosome assembly	83	142	1.82E-26
GO:0034728	nucleosome organization	89	182	3.11E-21
GO:0065004	protein-DNA complex assembly	89	207	1.33E-16
GO:0045455	ecdysteroid metabolic process	37	51	1.68E-15
GO:0071824	protein-DNA complex organization	95	247	4.77E-14
GO:0071103	DNA conformation change	102	275	6.46E-14
GO:0008202	steroid metabolic process	68	153	2.11E-13
GO:0042445	hormone metabolic process	71	164	2.18E-13
GO:0006338	chromatin remodeling	141	466	1.88E-11
GO:0042180	ketone metabolic process	44	90	6.15E-10
GO:0005984	disaccharide metabolic process	25	35	6.15E-10
GO:0000023	maltose metabolic process	19	22	8.92E-10
GO:0060568	regulation of peptide hormone processing	15	15	1.22E-09
GO:0060570	negative regulation of peptide hormone processing	15	15	1.22E-09
GO:0006865	amino acid transport	50	114	2.04E-09
GO:0006325	chromatin organization	175	663	6.08E-09
GO:0003333	amino acid transmembrane transport	44	98	1.43E-08
GO:0140718	facultative heterochromatin formation	25	39	1.50E-08
GO:0000183	rDNA heterochromatin formation	25	39	1.50E-08
GO:0016321	female meiosis chromosome segregation	30	55	4.81E-08
GO:0032989	cellular anatomical entity morphogenesis	75	852	4.81E-08
GO:0007608	sensory perception of smell	42	95	5.79E-08
GO:0090251	protein localization involved in establishment of planar polarity	20	28	6.59E-08
GO:0010817	regulation of hormone levels	110	379	1.27E-07
GO:0015031	protein transport	52	653	1.27E-07
GO:1905546	cellular response to phenylpropanoid	12	12	1.45E-07
GO:0071412	cellular response to genistein	12	12	1.45E-07
GO:0071413	cellular response to hydroxyisoflavone	12	12	1.45E-07
GO:0038111	interleukin-7-mediated signaling pathway	23	37	1.56E-07
GO:0031507	heterochromatin formation	53	140	1.84E-07
GO:1903318	negative regulation of protein maturation	25	43	1.84E-07
GO:0010955	negative regulation of protein processing	25	43	1.84E-07
GO:0042573	retinoic acid metabolic process	16	20	2.09E-07
GO:0009059	macromolecule biosynthetic process	224	1931	2.20E-07
GO:0015833	peptide transport	54	658	2.98E-07
GO:0090163	establishment of epithelial cell planar polarity	18	25	2.99E-07
GO:0007599	hemostasis	49	126	2.99E-07
GO:0050878	regulation of body fluid levels	73	222	3.03E-07
GO:0009311	oligosaccharide metabolic process	29	57	4.56E-07
GO:0050817	coagulation	48	124	4.65E-07

Supplementary Table S5 (continued). GO enrichment of expanded genes in *Er. hayati.*

GO	Description	Study	Pop	p_fdr
GO:0006335	DNA replication-dependent chromatin assembly	23	39	4.65E-07
GO:0098760	response to interleukin-7	23	39	4.65E-07
GO:0098761	cellular response to interleukin-7	23	39	4.65E-07
GO:0006959	humoral immune response	43	106	4.74E-07
GO:0033215	reductive iron assimilation	11	11	5.59E-07
GO:0010467	gene expression	168	1513	5.68E-07
GO:0006629	lipid metabolic process	161	642	8.17E-07
GO:0006412	translation	14	288	8.29E-07
GO:0033595	response to genistein	12	13	8.98E-07
GO:0033594	response to hydroxyisoflavone	12	13	8.98E-07
GO:0009653	anatomical structure morphogenesis	235	1977	9.27E-07
GO:0048646	anatomical structure formation involved in morphogenesis	74	800	1.24E-06
GO:0070828	heterochromatin organization	53	147	1.26E-06
GO:0046942	carboxylic acid transport	65	199	1.91E-06
GO:0015849	organic acid transport	65	199	1.91E-06
GO:0045814	negative regulation of gene expression, epigenetic	53	151	2.49E-06
GO:0048518	positive regulation of biological process	378	2926	3.08E-06
GO:0042537	benzene-containing compound metabolic process	16	23	3.67E-06
GO:0050678	regulation of epithelial cell proliferation	3	150	4.10E-06
GO:0043090	amino acid import	21	37	4.45E-06
GO:0005975	carbohydrate metabolic process	84	288	4.93E-06
GO:0019730	antimicrobial humoral response	33	77	5.28E-06
GO:0048468	cell development	219	1834	5.44E-06
GO:0010927	cellular component assembly involved in morphogenesis	4	161	6.24E-06
GO:0019748	secondary metabolic process	52	151	7.48E-06
GO:1905039	carboxylic acid transmembrane transport	48	134	7.61E-06
GO:0046907	intracellular transport	111	1056	7.66E-06
GO:0032351	negative regulation of hormone metabolic process	16	24	7.77E-06
GO:0019585	glucuronate metabolic process	14	19	7.77E-06
GO:0060541	respiratory system development	19	315	7.77E-06
GO:0043043	peptide biosynthetic process	18	305	1.04E-05
GO:0048869	cellular developmental process	314	2471	1.11E-05
GO:0048731	system development	367	2821	1.21E-05
GO:0006936	muscle contraction	80	96	1.21E-05
GO:0051290	protein heterotetramerization	23	46	1.82E-05
GO:0042381	hemolymph coagulation	10	11	1.87E-05
GO:1903964	monounsaturated fatty acid metabolic process	10	11	1.87E-05
GO:1903966	monounsaturated fatty acid biosynthetic process	10	11	1.87E-05
GO:0017001	antibiotic catabolic process	17	28	1.97E-05
GO:0045184	establishment of protein localization	69	720	2.48E-05

Supplementary Table S5 (continued). GO enrichment of expanded genes in *Er. hayati.*

GO	Description	Study	Pop	p_fdr
GO:0000902	cell morphogenesis	71	735	2.48E-05
GO:0050872	white fat cell differentiation	15	23	2.72E-05
GO:0044341	sodium-dependent phosphate transport	15	23	2.72E-05
GO:0048522	positive regulation of cellular process	365	2785	3.33E-05
GO:0071361	cellular response to ethanol	18	32	3.92E-05
GO:0006886	intracellular protein transport	45	524	4.32E-05
GO:0050830	defense response to Gram-positive bacterium	35	90	4.35E-05
GO:0080184	response to phenylpropanoid	12	16	4.36E-05
GO:0071394	cellular response to testosterone stimulus	12	16	4.36E-05
GO:0009058	biosynthetic process	342	2621	5.27E-05
GO:0051276	chromosome organization	187	821	5.43E-05
GO:1901655	cellular response to ketone	36	96	5.50E-05
GO:0042447	hormone catabolic process	15	24	5.50E-05
GO:0030154	cell differentiation	304	2367	5.68E-05
GO:0006721	terpenoid metabolic process	20	39	5.74E-05
GO:0034654	nucleobase-containing compound biosynthetic process	88	850	6.19E-05
GO:0007606	sensory perception of chemical stimulus	46	137	6.57E-05
GO:0006820	monoatomic anion transport	88	324	6.59E-05
GO:0033189	response to vitamin A	13	19	6.79E-05
GO:1903699	tarsal gland development	10	12	7.48E-05

* Study: GO count in the expanded genes set; Pop: GO count in all genes in the specie; p_fdr: corrected p-value using method FDR (BH). The table presents the top 100 data entries arranged in ascending order of p_fdr.

Supplementary Table S6. GO enrichment of contracted genes in *Er. hayati*, p_fdr < 0.05.

GO	Description	Study	Pop	p_fdr
GO:0032310	prostaglandin secretion	16	16	1.33E-20
GO:0140353	lipid export from cell	16	17	1.10E-19
GO:0015732	prostaglandin transport	16	19	3.94E-18
GO:0032309	icosanoid secretion	16	23	6.64E-16
GO:1901571	fatty acid derivative transport	16	26	8.78E-15
GO:0071715	icosanoid transport	16	26	8.78E-15
GO:0002576	platelet degranulation	18	46	2.25E-12
GO:0015908	fatty acid transport	17	46	3.83E-11
GO:0046717	acid secretion	16	49	1.87E-09
GO:0015718	monocarboxylic acid transport	20	85	2.48E-09
GO:0120036	plasma membrane bounded cell projection organization	69	921	4.21E-08
GO:0030030	cell projection organization	69	937	6.43E-08
GO:0040011	locomotion	68	1014	6.91E-06
GO:0042221	response to chemical	117	2197	8.42E-06
GO:0015698	inorganic anion transport	17	100	1.57E-05
GO:0006869	lipid transport	19	129	2.43E-05
GO:0023061	signal release	22	172	2.61E-05
GO:0006935	chemotaxis	36	418	5.46E-05
GO:0007411	axon guidance	31	337	0.000100986
GO:0086064	cell communication by electrical coupling involved in cardiac conduction	5	6	0.000127267
GO:0097485	neuron projection guidance	31	344	0.000144649
GO:0060271	cilium assembly	25	241	0.000153422
GO:0044782	cilium organization	25	243	0.0001719
GO:0010876	lipid localization	20	165	0.000189966
GO:0032501	multicellular organismal process	191	4419	0.000195148
GO:0003008	system process	60	958	0.000252947
GO:0051239	regulation of multicellular organismal process	95	1798	0.000309704
GO:1900194	negative regulation of oocyte maturation	5	7	0.000309704
GO:0007275	multicellular organism development	156	3441	0.000321504
GO:0034220	monoatomic ion transmembrane transport	38	497	0.000321504
GO:0048468	cell development	96	1834	0.000340303
GO:0032989	cellular anatomical entity morphogenesis	55	852	0.000345193
GO:0050877	nervous system process	48	710	0.000363476
GO:0030182	neuron differentiation	64	1060	0.000363476
GO:0061564	axon development	36	460	0.000365503
GO:0120031	plasma membrane bounded cell projection assembly	27	295	0.000382739
GO:0009605	response to external stimulus	79	1427	0.000442389
GO:0048856	anatomical structure development	163	3677	0.000525383
GO:0042330	taxis	36	472	0.000525383

Supplementary Table S6 (continued). GO enrichment of contracted genes in *Er. hayati.*

GO	Description	Study	Pop	p_fdr
GO:0048666	neuron development	56	898	0.000525383
GO:0060283	negative regulation of oocyte development	5	8	0.000525383
GO:0007366	periodic partitioning by pair rule gene	5	8	0.000525383
GO:0015849	organic acid transport	21	199	0.000525383
GO:0046942	carboxylic acid transport	21	199	0.000525383
GO:0045595	regulation of cell differentiation	65	1111	0.000525383
GO:0070925	organelle assembly	41	573	0.000637153
GO:0009653	anatomical structure morphogenesis	100	1977	0.000684269
GO:0048731	system development	131	2821	0.000833236
GO:0030031	cell projection assembly	27	314	0.00093083
GO:1905880	negative regulation of oogenesis	5	9	0.000966709
GO:0010644	cell communication by electrical coupling	5	9	0.000966709
GO:0016043	cellular component organization	154	3488	0.001151726
GO:0048667	cell morphogenesis involved in neuron differentiation	39	555	0.0013365
GO:0086065	cell communication involved in cardiac conduction	6	16	0.001566137
GO:0006811	monoatomic ion transport	42	636	0.002059149
GO:0032502	developmental process	165	3843	0.002383295
GO:0007409	axonogenesis	32	431	0.002383295
GO:0048513	animal organ development	105	2178	0.002508727
GO:0090257	regulation of muscle system process	16	141	0.002703769
GO:1903430	negative regulation of cell maturation	5	11	0.002855157
GO:0007623	circadian rhythm	16	142	0.002861648
GO:0098656	monoatomic anion transmembrane transport	21	227	0.002981919
GO:0050896	response to stimulus	160	3718	0.002981919
GO:0031175	neuron projection development	43	671	0.002981919
GO:0010045	response to nickel cation	4	6	0.002981919
GO:0009888	tissue development	78	1503	0.003028619
GO:0007600	sensory perception	29	376	0.003138622
GO:0007399	nervous system development	90	1804	0.00320789
GO:0052652	cyclic purine nucleotide metabolic process	7	27	0.00320789
GO:0009187	cyclic nucleotide metabolic process	7	27	0.00320789
GO:0010038	response to metal ion	22	250	0.003621752
GO:1900193	regulation of oocyte maturation	6	19	0.003664271
GO:0009410	response to xenobiotic stimulus	44	701	0.003757374
GO:0072359	circulatory system development	35	500	0.003761713
GO:0055085	transmembrane transport	41	633	0.003761713
GO:0010882	regulation of cardiac muscle contraction by calcium ion signaling	5	12	0.003761713
GO:0035239	tube morphogenesis	44	707	0.004103656
GO:0042752	regulation of circadian rhythm	14	118	0.004301155
GO:0045944	positive regulation of transcription by RNA polymerase II	41	643	0.004524493

Supplementary Table S6 (continued). GO enrichment of contracted genes in *Er. hayati.*

GO	Description	Study	Pop	p_fdr
GO:0030154	cell differentiation	110	2367	0.00473309
GO:0060538	skeletal muscle organ development	14	120	0.005011714
GO:0000902	cell morphogenesis	45	735	0.005048984
GO:0007168	receptor guanylyl cyclase signaling pathway	3	3	0.005048984
GO:0048699	generation of neurons	72	1384	0.005048984
GO:0046477	glycosylceramide catabolic process	4	7	0.005048984
GO:0071872	cellular response to epinephrine stimulus	4	7	0.005048984
GO:0071871	response to epinephrine	4	7	0.005048984
GO:0044057	regulation of system process	24	297	0.005048984
GO:0071840	cellular component organization or biogenesis	155	3632	0.00521633
GO:0031032	actomyosin structure organization	14	122	0.005434146
GO:0007417	central nervous system development	44	720	0.00620348
GO:0022008	neurogenesis	74	1447	0.006521221
GO:0035295	tube development	54	952	0.006659767
GO:0009887	animal organ morphogenesis	58	1051	0.006659767
GO:0006941	striated muscle contraction	8	42	0.006659767
GO:0048869	cellular developmental process	113	2471	0.006823265
GO:0015711	organic anion transport	22	266	0.006848703
GO:0050793	regulation of developmental process	81	1624	0.007175485
GO:0051093	negative regulation of developmental process	38	588	0.007371847
GO:0001952	regulation of cell-matrix adhesion	9	55	0.007581484
GO:0061061	muscle structure development	30	425	0.007581484

* Study: GO count in the expanded genes set; Pop: GO count in all genes in the specie; p_fdr: corrected p-value using method FDR (BH). The table presents the top 100 data entries arranged in ascending order of p_fdr.

Supplementary Table S7. The proportion of various transposons in genome sequence in some Hymenoptera species.

Species	Transposable elements contents (%)					Genome size (Mb)
	DNA	LTR	nonLTR	Unknown	Total	
Athalia rosae	2.93	0.32	0	0.85	4.1	169
Orussus abietinus	8.98	5.07	0.03	3.25	17.33	186
Trichogramma pretiosum	2.96	1.61	0.03	1.5	6.1	188
Cotesia chilonis	11.6	7.47	0.78	8.13	27.98	190
Polistes dominula	3.23	0.89	0.54	5.29	9.95	208
Ooceraea biroi	13.66	17.88	1.51	5.6	38.65	218
Apis mellifera	1.23	0.16	0	2.99	4.38	227
Ceratosolen solmsi	0.93	0.13	0	1.49	2.55	277
Nasonia vitripennis	12.38	10.82	0	0	23.2	297
Atta cephalotes	10.1	0.35	0.39	7.81	18.65	317
Bombus terrestris	4.01	0.72	0.45	2.51	7.68	321
Pteromalus puparum	16.66	14.87	1.01	6.1	38.64	338
Encarsia formosa	18.58	18.76	0	0.78	38.12	452
Copidosoma floridanum	12.64	11.63	0.59	9.31	34.17	554
Gonatopus flavifemur	40.14	8.2	0.45	4.48	53.27	637
Eretmocerus hayati	23.34	19.44	0.92	6.51	50.19	692
Belonocnema treatae	43.56	6.69	0.64	9.72	60.61	1558
